# Is an irritable ADHD profile traceable using personality dimensions? Replicability, stability, and predictive value over time of data-driven profiles

**DOI:** 10.1007/s00787-020-01546-z

**Published:** 2020-05-12

**Authors:** Tessa F. Blanken, Ophélie Courbet, Nathalie Franc, Ariadna Albajara Sáenz, Eus J.W. Van Someren, Philippe Peigneux, Thomas Villemonteix

**Affiliations:** 1 Department of Sleep and Cognition, Netherlands Institute for Neuroscience, Meibergdreef 47, 1105 BA Amsterdam, The Netherlands; 2 Psychopathology and Neuropsychology Lab, Paris 8 University, Rue de la Liberté 2, 93526 Saint-Denis, France; 3 Médecine Psychologique de L’enfant Et de L’adolescent (MPEA1), MPEA Secteur 1, Hôpital Saint-Éloi, CHU de Montpellier, 80 avenue Augustin-Fliche, 34295 Montpellier, France; 4 Neuropsychology and Functional Neuroimaging Research Unit at CRCN–Center for Research in Cognition and Neurosciences and UN-ULB Neurosciences Institute, Université Libre de Bruxelles (ULB), CP191 Avenue Franklin Roosevelt 50, 1050 Brussels, Belgium

**Keywords:** ADHD, Heterogeneity, Irritability, Dispositional traits, Personality traits, Community detection

## Abstract

Pediatric attention deficit/hyperactivity disorder (ADHD) is a heterogeneous condition. In particular, children with ADHD display varying profiles of dispositional traits, as assessed through temperament and personality questionnaires. Previous data-driven community detection analyses based on temperament dimensions identified an irritable profile of patients with ADHD, uniquely characterized by elevated emotional dysregulation symptoms. Belonging to this profile increased the risk of developing comorbid disorders. Here, we investigated whether we could replicate this profile in a sample of 178 children with ADHD, using community detection based on personality dimensions. Stability of the identified profiles, of individual classifications, and clinical prediction were longitudinally assessed over a 1-year interval. Three personality profiles were detected: The first two profiles had high levels of neuroticism, with the first displaying higher ADHD severity and lower openness to experience (profile 1; *N* = 38), and the second lower agreeableness (profile 2; *N* = 73). The third profile displayed scores closer to the normative range on all five factors (profile 3; *N* = 67). The identified profiles did only partially replicate the temperament-based profiles previously reported, as higher levels of neuroticism were found in two of the three detected profiles. Nonetheless, despite changes in individual classifications, the profiles themselves were highly stable over time and of clinical predictive value. Whereas children belonging to profiles 1 and 2 benefited from starting medication, children in profile 3 did not. Hence, belonging to an emotionally dysregulated profile at baseline predicted the effect of medication at follow-up over and above initial ADHD symptom severity. This finding suggests that personality profiles could play a role in predicting treatment response in ADHD.

**Electronic supplementary material:**

The online version of this article (10.1007/s00787-020-01546-z) contains supplementary material, which is available to authorized users.

## Introduction

Heterogeneity in psychiatric disorders as defined by current nosologies such as the DSM-5 is ubiquitous. Most disorders can indicate multiple, only partially overlapping, symptom profiles that are likely to result from multiple independent mechanistic pathways [1–6]. A prominent example is attention deficit/hyperactivity disorder (ADHD), a diagnosis associated with multiple risk factors [3], a range of comorbidities [7], and various impairments [8, 9]. Heterogeneity in ADHD is evident across multiple levels: from genetics [10] to neural systems [11, 12], cognition [13–15], and clinical course [15]. This multi-level heterogeneity has hampered the quest for neurobiological markers [11] and the optimization of treatment. Finding meaningful ways to address heterogeneity when assessing etiological factors, neurobiological profiles, and clinical outcomes is, therefore, crucial.

In the DSM-5, ADHD is defined as a neurodevelopmental syndrome with two main symptom domains of inattentive and hyperactive/impulsive symptoms [16]. Next to these core symptoms, ADHD is consistently associated with dispositional traits, i.e., stable individual differences in human behaviour as assessed through temperament and personality questionnaires [17, 18]. Specifically, children and adults with ADHD are on average lower in conscientiousness or effortful control, reactive control, and agreeableness, and higher on neuroticism or negative emotionality, when compared with typically developing individuals [19–21]. At the same time, children with ADHD show differential patterns in these dispositional traits that are potentially clinically relevant [22]. Using clustering techniques to uncover different profiles of dispositional traits might, therefore, offer a promising approach to uncover within-diagnosis profiles that potentially have unique clinical predictive value [23].

In a pioneering study on heterogeneity in ADHD, Karalunas et al. [12] used community detection [24] on dispositional traits (i.e., temperament scores) to detect biologically informed profiles [13]. Three novel data-driven temperament profiles were identified within ADHD, which were labelled mild, surgent and irritable; each associated with distinct neurobiological correlates. The three profiles were replicated in an independent sample, and the irritable profile was shown to be the most stable over time, as 61% of irritable children were consistently assigned to the same profile across all 3 years of follow-up [25]. Importantly, in a combined model including initial ADHD presentation (i.e., predominantly inattentive, predominantly hyperactive/impulsive, or combined) and oppositional defiant disorder (ODD) diagnosis, only the identified temperament profiles were found to predict the onset of new disorders. Crucially, the irritable profile consisted of children both with and without comorbid diagnoses, and 65% of the children that belonged to this group were free of any comorbid diagnosis, including ODD or disruptive mood dysregulation disorder (DMDD). This stresses the unique clinical relevance of the temperament profiles, when compared with existing diagnoses and subtypes.

Because of the high stability and unique clinical predictive value, this irritable profile is of particular interest. Irritability designates a proneness to anger that is inconsistent with an individual's developmental level [26] and is best described by two components: tonic and phasic. The tonic component refers to a persistent angry, grumpy, or grouchy mood, whereas the phasic component refers to behavioural outbursts of intense anger [27]. Although irritability is not a defining diagnostic feature, impairments in irritability are highly prevalent in childhood ADHD and affect at least half of patients in clinical samples [28–30]. Irritability is also strongly associated to defiant behaviours [31], and as such defined as a dimension of ODD in the DSM-5 [16].

Next to increased levels of anger, the irritable profile identified by Karalunas et al. [12, 25] was also characterized by broader emotional dysregulation (ED) manifestations such as discomfort, fear, and sadness. The finding that the irritable profile predicted new onset of comorbid disorders (primarily in the form of new anxiety disorders) in patients with ADHD is in line with available data, showing that the irritable dimension of ODD is a significant predictor of depression and anxiety disorders [32]. In clinical practice, investigating whether a child belongs to the irritable profile may, therefore, help to identify a subgroup of patients with ADHD with sub-diagnostic ED manifestations who are at increased risk of developing emotional comorbidities, which opens up new possibilities for early detection and prevention.

For the identified irritable profile to fulfil this potential, reproducibility across samples is essential. Karalunas et al. [25] replicated their initial findings in a new sample, but both samples were collected within the Oregon ADHD Program in the United States. Whether the three profiles, and in particular, the irritable profile can also be found in other cultural areas remains to be established. Furthermore, dispositional traits were exclusively assessed using the Temperament in Middle Childhood Questionnaire (TMCQ)—a theory-driven questionnaire based on Rothbart’s temperament model [33]. Yet, it should be noted that the best way to conceptualize dispositional traits in children remains a matter of disagreement in the field, and multiple trait-based personality and temperament models coexist [23].

Historically, temperament and personality traits were studied separately. Compared to personality traits, temperament was thought to capture traits that have a stronger genetic or neurobiological basis [34]. In Rothbart’s model, the trait structure is summarized in three broad, well-validated domains: (a) negative affect, encompassing emotions such as fear, sadness, and anger/frustration; (b) positive affect (or surgency), reflecting tendency to express excitement and happiness, willingness to approach novel stimuli, and overall activity level; and (c) effortful control, describing top down self-regulatory capacities and tendencies [34].

Despite the initial coexistence of these two separate lines of research, several scholars made a compelling case that temperament and personality systems describing children’s and adolescent’s traits can be considered to be “more alike than different”, and are actually tapping into overlapping trait domains in somewhat different fashions [18, 34–38]. In this context, the most common personality model, the five factor model (FFM), has been described as a useful unifying framework [36]. Previous research indeed suggests that the dimensions of neuroticism (predisposed to emotional distress vs. emotionally stable), extraversion (energic and thrill-seeking vs. sober and solitary), and conscientiousness (disciplined and fastidious vs. laid-back and careless) represent the three higher order domains of negative affect, positive affect, and effortful control found in the Rothbart’s temperament model [36]. In addition, the FFM represents the dimensions of agreeableness (kind and trusting vs. competitive and arrogant) and openness to experience (curious and unconventional vs. traditional and pragmatic), whereas these two traits were only measured as subscales in Rothbart’s temperament framework [36].

It remains currently unclear if the identification of a robust and clinically relevant irritable profile in ADHD depends on the dispositional framework (i.e., personality or temperament) that is used. Do both frameworks yield overlapping profiles, or does each framework provide unique information resulting in different profiles? One previous study by Martel et al. [23] examined data-driven profiles based on the Big Five personality factors in a large sample of children and adolescents with and without ADHD, and identified six different profiles [23]. Although some similarities between these profiles and those identified by Karalunas et al. can be found, they cannot be easily matched. For example, high neuroticism or negative emotionality was not specific to one but two profiles, and this was not the dimension along which the profiles differed the most. These results suggest that despite potentially tapping into the same construct, temperament and personality questionnaires may contribute to identify different profiles in children and adolescents with ADHD, possibly yielding each unique predictive value. Another possibility for failing to replicate the ADHD profiles across dispositional frameworks might be that both samples differ in age range (7–11 years vs. 6–18 years). Age-related effects on temperament and personality have indeed been solidly established during childhood and adolescence [36, 39].

In the present study, we, therefore, investigated whether the profiles identified by Karalunas et al. [12, 25] are robust across dispositional traits by examining their replicability in a new sample of children with ADHD with a similar age range, using a different measure of dispositional traits (i.e., a personality questionnaire). A sample of children with combined-type ADHD was recruited, and assessed at two time points with a 1-year interval. This approach allowed us to concurrently investigate the temporal stability of both the profiles and the classification of individuals. Children were assessed with the Big Five questionnaire for children (BFQ-C), a tool instantiating the FFM, with an empirically well-established five factor structure [40–43]. Measures of clinical severity were also obtained, and the predictive value of the profiles identified at T0 with regard to severity at T1 was investigated. We hypothesized that the neuroticism items of the FFM would drive the detection of a subgroup of children with ADHD characterized by unique increases in irritability, and that this subgroup would be the most stable over time.

## Methods

### Recruitment procedure

221 French-speaking families living in France or Belgium participated in this study. The research was approved by the Ethics Committee of the ULB-Erasme Hospital, Brussels, Belgium (P2016/124) and, therefore, conducted in accordance with the latest version of the Declaration of Helsinki. Families were recruited through a network of clinicians (52% of final cases), and by advertising through the Belgian and French national ADHD associations who advertised this research project on their social network webpages (48%). Child psychiatrists, pediatricians, and child psychologists belonging to an informal network of ADHD researchers affiliated to the French-Speaking ADHD International Congress were contacted by the last author to assist with recruitment. Clinicians introduced the research design to parents and provided them with a booklet describing the project along with contact information. Families volunteered through emails, and were contacted by the research team through telephone to assess eligibility. Children had to be 6–11 years old, to have been diagnosed with ADHD, and to be medication naïve at the time of recruitment. Parents were each invited to fill in the questionnaires online, while being informed that one informant was sufficient to participate in the study. A phone interview was scheduled with one parent to collect demographic information and to conduct a diagnostic interview. One year after the initial (T0) phone interview, parents were contacted by email and provided with codes for follow-up assessment (T1). At each time point, access to the interface was preceded by an information statement and conditioned to the validation of an electronic consent form.

### Diagnostic procedure and exclusion process

After eligibility assessment, one parent completed a semi-structured clinical interview (Kiddie Schedule for Affective Disorders and Schizophrenia for School Aged Children-Present and Lifetime Version (K-SADS-PL); [44]) administered during a phone call by a doctorate-level clinical psychologist [TV] with a 10-year practice experience in child psychiatry. Participants were excluded if they failed to meet the diagnostic criteria for combined-type ADHD based on the K-SADS-PL; were prescribed psychotropic medications, had neurological impairment, seizure history, other major medical conditions, prior diagnosis of intellectual disability, autism spectrum disorder (ASD), or psychosis. Participants were further excluded if their score at the ADHD Rating Scale IV (ADHD-RS-IV) total scale was below the 93th percentile for their age group [45]. Of the 221 families that were approached initially, 43 were excluded: 20 failed to complete all questionnaires at T0; 16 children were excluded based on the clinical interview, which revealed insufficient symptom numbers or clinical severity, or a non-combined form of ADHD; 4 children were excluded due the presence of comorbid ASD; finally, 3 participants were excluded based on their ADHD-RS Total scores. The final sample at the first time point (T0), therefore, consisted of 178 combined-type ADHD cases. Demographic characteristics of the sample are reported in Table 1. At T1, 12 families failed to complete the second wave of questionnaires, yielding a follow-up sample of 166 cases (attrition rate: 6.7%).

**Table 1:** Descriptive information and longitudinal outcomes for the whole sample

Characteristics	T0	T1
Basic demographics		
*N*	178	166
(Boys: girls)	(135: 43)	(126: 40)
Age mean (sd) years	8.2 (1.4)	9.2 (1.4)
*N* (%) on stimulant medication	0 (0)	74 (44,6)
Comorbidity (%)		
GAD	16.9	
Specific phobia	4.5	
SAD	2.8	
Social phobia	1.1	
Enuresia	2.8	
Encopresia	1.1	
Tic disorder	5.1	
ODD	5.6	
DMDD (disruptive mood dysregulation disorder)	13.5	
ADHD and severity measures	*M* (SD)	*M* (SD)
ADHD-RS total score	41.20 (6.76)	36.05 (9.32)
ADHD-RS inattentive score	20.61 (4.11)	18.21 (4.91)
ADHD-RS Hyp/imp score	20.59 (4.22)	17.84 (5.52)
SDQ impact score	6.01 (2.59)	5.11 (2.77)

*GAD* generalised anxiety disorder, *SAD* separation anxiety disorder, *ODD* oppositional defiant disorder, *DMDD* disruptive mood dysregulation disorder, *ADHD-RS* attention deficit with hyperactivity rating scale IV, *SDQ* strengths and difficulties questionnaire, *T0* time 0, *T1* time 1

### Assessment of personality and clinical outcomes

At least one parent of each child completed the Big Five questionnaire for children (BFQ-C) at each time point [40] (French adaptation: [43]). The BFQ-C is based on the FFM, and contains a total of 65 items, with five scales of 13 items. At T0, double informant data (i.e., mother and father data) were available for 15 (8.4%) of the initial 178 children participating. For these cases, scores were averaged across informants at the item level prior to any reported analysis.^1^^1^ For single informant data, the same informant completed the questionnaire at both time points. Scale reliabilities were assessed at T0, yielding the following (Cronbach’s alpha) scale reliabilities: 0.71 for extraversion, 0.85 for agreeableness, 0.81 for conscientiousness, 0.83 for neuroticism, and 0.84 for openness.

Clinical outcome was evaluated using scores of parent-rated functional impairment on the Impact Supplement of the Strengths and Difficulties Questionnaire (SDQ) for age 4–17. Items on overall distress and impairment were summed to generate an impact score ranging from 0 to 10, with higher scores indicating greater impact [46, 47].

### Statistical analysis

To identify communities, we followed the same procedures as Karalunas et al. [12, 25].

#### Data preparation

We first standardized the 65 items of the BFQ-C to the sample mean and standard deviation, after which we computed the child-by-child profile correlations.

#### Community detection

We applied the weight conserving modularity algorithm to the child-by-child correlation matrix [24, 48]. Initially, the algorithm places each child (i.e., node) into its own community. In subsequent steps, communities are reassigned until a division of the network into communities is made for which the modularity is optimized. The modularity index *Q* is a metric that quantifies the quality of the placing of nodes into communities, with higher values indicating better partitioning of the data into communities. In practice, most values of *Q* fall between 0.3 and 0.7, with values close to 0.3 reflecting weakly defined communities, and values around 0.7 reflecting strong community structures [49]. We used an adapted version of the modularity to take the sign of the weight into account, as we assumed that both positive and negative weights are informative of the similarities and differences between children. Positive weights indicate that two children have similar scoring patterns, and thus provide support that these children should be in similar communities. The more similar the scoring pattern, the higher the correlation, and the stronger the support for two children to belong to the same community (reflected in *Q*^+^). Negative weights, on the other hand, indicate that two children have opposite scoring patterns. An opposite scoring pattern might indicate important qualitative differences and should thus provide support for children to belong to different communities (reflected in *Q*^−^). Because the community detection algorithm is not deterministic, the optimal number of communities and associated modularity (*Q*) can differ slightly with different runs. To obtain stable results, the final assignment of children was based on the modal group assignment across ten runs.

To improve comparability with Martel et al. [23] and as done by Karalunas et al. [25], we also conducted a latent profile analysis based on the scores at the 65 items of the BFQ-C. The methodology and results are presented in the Supplement.

#### Interpretation and representation

We compared the identified communities on the personality factors using analysis of variance (ANOVA) tests. Post hoc tests with Scheffé corrections were performed to determine significant group differences. We visualized the different communities by their patterns on the five personality factors as assessed with the BFQ-C. Moreover, to interpret the communities at a more detailed level, we conducted an exploratory factor analysis on the BFQ-C items. This allowed us to characterize the identified communities at a more detailed personality facet level, similar to Karalunas et al. [12, 25] who used the 16 more detailed temperament subscales instead of the three higher order factors. Details on the exploratory factor analysis are given in the Supplementary Material.

#### Temporal stability

We assessed the stability of the identified communities over time and compared the communities estimated at T0 to the communities at T1. We compared both the communities themselves (i.e., community-profile stability) as well as whether children were consistently assigned to the same community (i.e., community-membership stability). In addition, we performed a multinomial logistic regression to assess the community-membership stability.

#### Stability across measurement levels

Finally, we explored whether the identified communities depend on the measurement level on which the child-by-child correlation matrix was built. We originally estimated the correlation among children based on their scoring pattern on the 65 individual items of the BFQ-C. Alternatively, one could estimate the correlation among children based on their scoring pattern on the five personality factors. We applied the weight conserving modularity algorithm to both matrices and evaluated the stability of the identified communities by computing the correlation between corresponding communities and computing their mean absolute difference.

Note that the community detection algorithm is based on the child-by-child correlation matrix, and that the dimensions of this matrix are the same, regardless of using 65 items or 5 factors to compute the correlation between two children. Thus, regardless of the numbers of features used to correlate the scoring pattern of two children, computationally the input for the community detection remains the same (i.e., an *N* × *N* child-by-child correlation matrix).

#### Clinical prediction

We evaluated whether the identified communities differed in clinical outcome at T1 as assessed using the SDQ Impact score and the ADHD-RS total score. We first assessed whether the clinical outcomes differed over time using dependent *t* tests. Second, we evaluated whether each clinical outcome at T1 could be predicted by community membership, when controlling for baseline SDQ Impact score, baseline ADHD-RS total score, age, and sex. Third, whereas all children were medication free at baseline, some started treatment after entering into the study. We, therefore, also evaluated whether the clinical outcomes at T1 were predicted by treatment (yes vs. no), while controlling for baseline SDQ Impact score, baseline ADHD-RS total score, age, and sex. Finally, we explored whether a possible effect of medication on ADHD-RS total score would differ across the communities.

The analyses were conducted in SPSS (version 20) and R (version 3.5.2) using the package ‘psych’ (version 1.8.12).

## Results

### Personality profiles

**Fig. 1:**
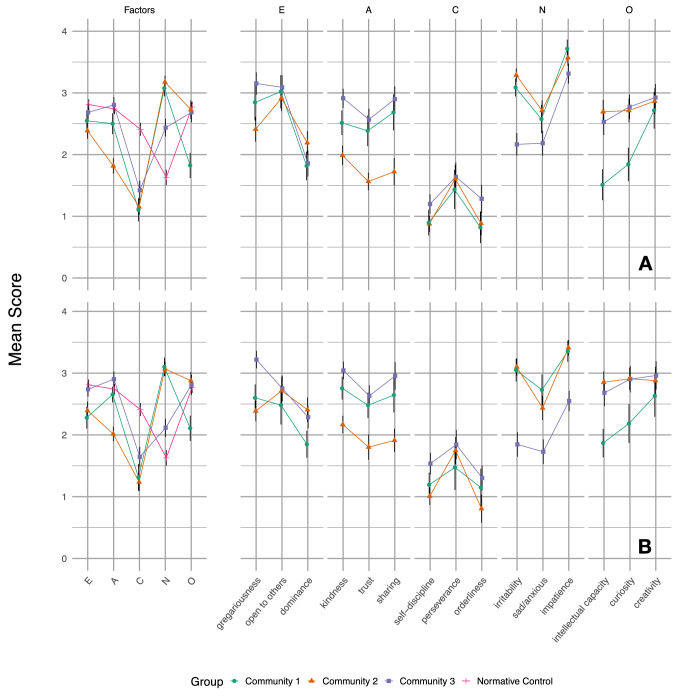
Profiles identified at baseline (top) and follow-up (bottom) using the 65 items. Error bars show 95% confidence intervals. *E* Extraversion, *A* agreeableness, *C* conscientiousness, *N* neuroticism, *O* openness to experience. Note that when interpreting the profiles, only for the five factors were normative data available. The scores on the subscales are shown to get a more detailed understanding of the different profiles, but should be interpreted with caution as no normative data are available

Community detection identified three communities at baseline of 38 (21%), 73 (41%) and 67 (38%) children. The average *Q* of 0.41 (range 0.41–0.42) across the 10 runs indicates moderate separation of the three communities. The identified profiles are visualized in Fig. 1a, and the descriptive information is given in Table 2. For reference, we plotted the profiles next to the average personality factor scores in a normative French sample that was kindly provided to us by Olivier and Hervé [43]. The scores in the normative French sample are also included in Supplementary Table 2.

**Table 2:** Characteristics of personality profiles

Characteristics	Time	Type 1	Type 2	Type 3	F(2,175)	Post hoc
Basic demographics						
* N*	T0	38	73	67		
	T1	38	66	62		
(Boys:Girls)	T0	(26:12)	(55:18)	(54:13)	χ^2^ (2) = 1.98	
	T1	(22:16)	(53:13)	(51:11)	χ^2^ (2) = 8.81*	
Age mean (sd) years	T0	8.5 (1.5)	8.1 (1.3)	8.2 (1.3)	1.40	
	T1	8.8 (1.4)	8.1 (1.4)	7.9 (1.2)	6.5**	1 > 2.3
*N* (%) on stimulant medication	T0	0 (0)	0 (0)	0 (0)		
	T1	20 (52,6)	32 (48,4)	40 (64,5)	χ^2^ (2) = 3.48	
Comorbidity (%, at T0)						
GAD		15.8	17.8	16.4	χ^2^ (2) = 0.09	
Specific phobia		0.0	9.6	1.5	χ^2^ (2) = 7.61*	
SAD		5.3	1.4	3.0	χ^2^ (2) = 1.40	
Social phobia		0.0	2.7	0.0	χ^2^ (2) = 2.91	
Enuresia		2.6	1.4	4.7	χ^2^ (2) = 1.24	
Encopresia		0.0	2.7	0.0	χ^2^ (2) = 2.91	
Tic disorder		5.3	5.5	4.5	χ^2^ (2) = 0.08	
ODD		5.3	9.6	1.5	χ^2^ (2) = 4.33	
DMDD		13.1	20.5	6.0	χ^2^ (2) = 6.37*	
ADHD and severity measures		*M* (SD)	*M* (SD)	*M* (SD)		
ADHD-RS total score	T0	43.47 (6.28)	41.72 (6.46)	39.35 (6.98)	5.09*	1 > 3
	T1	40.76 (8.21)	40.34 (6.16)	42.50 (6.26)	1.79	
ADHD-RS inattentive score	T0	22.53 (3.70)	20.26 (4.03)	19.90 (4.15)	5.68*	1 > 2.3
	T1	21.37 (4.11)	19.71 (4.42)	20.91 (3.75)	2.33	
ADHD-RS Hyp/imp score	T0	20.95 (4.26)	21.46 (3.61)	19.46 (4.61)	4.25*	2 > 3
	T1	19.39 (5.45)	20.62 (3.33)	21.59 (3.67)	3.54*	3 > 1
SDQ impact score	T0	6.07 (2.64)	6.65 (2.47)	5.28 (2.53)	5.15*	2 > 3
	T1	5.86 (2.5)	4.12 (2.77)	5.69 (2.65)	7.53**	1 > 2

*GAD* generalised anxiety disorder, *SAD* separation anxiety disorder, *ODD* oppositional defiant disorder, *DMDD* disruptive mood dysregulation disorder, *ADHD-RS* attention deficit with hyperactivity rating scale IV, *SDQ* strengths and difficulties questionnaire

* *p* < 0.05; ** *p* <  0.01

In terms of personality features, the first two profiles were characterized by high levels of neuroticism and low levels of conscientiousness when compared with the normative controls. Differences between these two profiles emerged on openness to experience and agreeableness, with the first profile displaying decreased levels of openness to experience when compared with the other groups, and the second profile displaying decreased levels of agreeableness when compared with the other groups. Finally, profile 3 displayed scores closer to the normative range on all five factors.

Clinically, profile 1 children were significantly more inattentive than the other two types, and displayed higher scores at the global ADHD-RS score than profile 3 children. Profile 2 children displayed significantly higher hyperactive/impulsive score than profile 3 children. Finally, profile 3 children presented the lowest ADHD-RS total score.

### Temporal stability

At follow-up, again three communities were identified, with *Q* = 0.44 (range 0.44–0.45), see Fig. 1b. The three communities identified at baseline resembled the identified communities at follow-up well: the correlation between the communities was lowest for community 3 (*r* = 0.89) and high for community 1 (*r* = 0.96) and 2 (*r* = 0.98). This was also reflected in the mean absolute difference across the communities, which was highest for community 3 (0.22 ± 0.19) and community 1 (0.21 ± 0.14), and lowest for community 2 (0.15 ± 0.07). This indicates that the communities are relatively stable over time at a group level. Big Five personality scores and subscale’s score for the three groups at T0 and T1 are presented in Supplementary Table S3.

At an individual level, the community membership was more stable than chance (*χ*^2^ = 67.97, *p* < 0.001). Overall, 63.8% of the children remained in the same community over time. Stability was highest for children in community 2 (71.0% remained) and 3 (65.0% remained), and lowest for children in community 1 (47.1% remained).

### Stability across measurement levels

Using the five personality factors instead of the items, again three communities were identified, with *Q* = 0.47 (range 0.46–0.48), see Supplementary Fig. 1 for the profiles. The three communities that were identified using either the 65 items or the 5 factors resembled each other very well: the correlation among the personality profiles ranged from 0.99–1.00, and the mean absolute difference ranged from 0.05–0.07 (SD range 0.05–0.06). The high correlations and small differences indicate that the information that is captured in the different items is highly similar compared with the information that is captured in the higher order factors.

### Clinical prediction

Parent-reported clinical impairment, measured by the SDQ Impact score, decreased significantly from baseline (6.0 ± 2.6 mean ± SD) to follow-up (5.1 ± 2.8 mean ± SD), *t *(165) = 4.09, *p* < 0.001. Clinical impairment at follow-up was predicted only by baseline clinical impairment (*t* = 5.7, *p* < 0.001).

ADHD severity, measured by the ADHD-RS total score, decreased significantly from baseline to follow-up, *t *(165) = 8.05, *p* < 0.001. Interestingly, ADHD severity at follow-up was predicted by community membership at baseline over and above baseline ADHD-RS total score, baseline SDQ Impact score, age, and sex. Specifically, the decrease in ADHD-RS total score for children in community 1 (− 9.3 ± 8.6 mean ± SD) was significantly larger than the decrease for both children in community 2 (− 4.7 ± 7.5 mean ± SD, *t* = 2.06, *p* = 0.04) and for children in community 3 (− 3.5 ± 8.5 mean ± SD, *t* = 2.37, *p* = 0.02). The decrease in ADHD-RS total score did not differ for children in community 2 compared to children in community 3 (*t* = 0.47, *p* = 0.64).

Between baseline and follow-up, almost half of the children (44.6%) had started medication treatment. We explored whether the start of medication might have affected the clinical outcomes at follow-up. While medication did not predict SDQ Impact score at follow-up, it did predict ADHD severity (*t* = − 2.40, *p* = 0.02), such that the decrease in ADHD-RS total score was, on average, higher for children who received medication (− 7.1 ± 8.9 mean ± SD) compared with children who did not receive medication (− 3.6 ± 7.6 mean ± SD).

Because both community membership and medication predicted ADHD severity at follow-up, we next explored whether the children that received medication were equally distributed across the different identified communities. Note that at baseline, the assessment used to identify the communities, none of the children received medication. Hence, there is no a priori reason to expect that medication and the communities are associated. The percentage of children that received medication was 61.8%, 39.1%, and 41.3% for communities 1–3, respectively (*χ*^2^ = 5.2, *p* = 0.08). Although the difference is non-significant, the relatively high percentage of children that received medication in the first community might explain the larger decrease in ADHD-RS total score in community 1 compared to both other communities.

Therefore, we evaluated whether ADHD severity at follow-up was predicted by community membership, medication use, or their interaction, while controlling for baseline ADHD-RS total score, baseline SDQ Impact score, age, and sex. There was a main effect for medication (*t* = − 2.6, *p* = 0.009), but not for community membership (*t* < 1.1, *p* > 0.26). There was, however, an interaction effect indicating that the effect of medication on ADHD severity in community 1 and community 2 was different compared with community 3 (*t* = 2.77, *p* = 0.006 and *t* = 2.55, *p* = 0.01, respectively). Specifically, medication use was associated to a larger decrease in ADHD-RS total score for children in community 1 (− 12.5 ± 8.4 mean ± SD for *N* = 21 medically treated children versus − 4.1 ± 6.0 mean ± SD for *N* = 21 non-medically treated children) and 2 (− 7.6 ± 7.7 mean ± SD for *N* = 27 medically treated children versus − 2.8 ± 6.8 mean ± SD for *N* = 42 non-medically treated children), while this was not the case for children in community 3 (− 2.4 ± 7.9 mean ± SD for *N* = 26 medically treated children versus − 4.4 ± 8.8 mean ± SD for *N* = 37 non-medically treated children). The effect of medication on ADHD-RS total score did not differ for children in community 1 or 2. This result suggests that the effectiveness of medication treatment might differ for different personality profiles in children with ADHD.

Because the different communities had different ADHD severity at baseline (i.e., 43.47 ± 6.28, 41.72 ± 6.46, and 39.35 ± 6.98 mean ± SD for communities 1–3, respectively), we considered the possibility that these baseline differences might confound our finding that medication did not affect children in this third community. To address this issue, we computed the mean ADHD-RS score at baseline for the children who completed both measurements (*N* = 166; 41.24 ± 6.76 mean ± SD) and selected only the children in the third community whose ADHD-RS total score was below one standard deviation of that mean (i.e., a score below 34.48). This resulted in selecting 52 of the 67 (77.6%) children, who had a mean ADHD-RS total score at baseline that was similar to that in community 1 and 2 (42.14 ± 5.01 mean ± SD). Using only this restricted sample, we re-evaluated our predictive model and still found a significant medication times community interaction effect, indicating that the effect of medication on children in community 3 was different from the children in community 1 and 2 (both *t* > 1.97, both *p* < 0.05).

## Discussion

In the present study, we set out to replicate the ADHD profiles detected in previous research by Karalunas et al. [12, 25] in a new sample of children with combined-typed ADHD, using a different measure of dispositional traits. In line with Karalunas et al. [12, 25], the optimal clustering solution yielded three different profiles, and the modularity index robustly stayed in the 0.4 range, reflecting a moderately defined community structure [49]. The personality profiles themselves were highly stable over time, despite changes in individual classification results.

While we also identified three profiles, we did not identify a unique profile characterized by emotional lability—labelled as the “irritable profile” by Karalunas et al. [12, 25]. Instead, two profiles presented with similarly increased levels of neuroticism when compared to normative controls (profiles 1 and 2), while differing on openness to experience and agreeableness levels. Of note, profile 3 resembled the “mild” profile identified in Karalunas et al. [12], being characterized by more normative scores on the neuroticism and conscientiousness scales, as well as by lower ADHD total scores. Finally, the "surgent" ADHD profile characterized by severe impulsivity, elevated assertiveness/dominance, and high-intensity pleasure seeking was not replicated.

Important differences in sample characteristics may have contributed to these partly divergent findings. First, Karalunas et al. [25] ADHD sample initially included 26% of children with a purely inattentive ADHD presentation, whereas our sample was exclusively composed of combined-type ADHD cases. Consequently, the profiles identified by Karalunas et al. may in part, reflect the different types of ADHD cases. This idea is supported by significant differences in the proportions of inattentive and combined types in the different groups identified in their sample [inattentive:combined (% inattentive); mild: 54:30 (64%), surgent: 15:119 (11%), irritable 27:110 (20%)]. Current inattentive ADHD cases may include in particular children with sluggish cognitive tempo, a possibly new and different clinical entity, which complexifies the interpretation of findings in mixed ADHD subtypes groups [50, 51]. Second, samples were recruited in different geographical areas (USA vs. France), and differences in dispositional traits have been reported in previous research investigating cross-cultural differences in children, with a tendency for lower levels of emotional stability in American vs. European children [52, 53]. In this context, whether stable temperamental or personality profiles should be expected in ADHD across different cultures remains an open question. Third, our initial sample was unmedicated, whereas 37% of Karalunas et al. [12] initial sample was taking stimulant medication for ADHD. Fourth, samples were recruited through different procedures: whereas our recruitment procedure required participants to have received a prior formal ADHD diagnosis by a clinician, Karalunas et al. [12, 25] research was based on the Oregon ADHD program, which recruited volunteers using mass mailing to parents in the local school districts, public advertisements, and community outreach to local clinics without such requirement. A bias in our case towards more clinic-referred compared with more community-referred cases in Karalunas’ et al. samples may have resulted in sample differences in ED levels. Indeed, while comorbidity rates were comparable (present sample: anxiety disorders 19.6%, ODD 5.6%, CD 0%, DMDD 13.5%; Karalunas et al. [25]: anxiety disorders 20.6%, ODD 19.3%, CD 1.9%, DMDD 4%), our sample appears to be more disturbed in the emotional domain (DMDD 13.5% vs 4%) but less oppositional/defiant (ODD 5.6% vs. 19.3%).

Alternatively, the differences between the identified profiles might have resulted from the differences in the measurement instrument (BFQ-C versus TMCQ) and underlying theoretical model (five factor model versus temperament model). Importantly, a recent study investigated the 16 subscales of the TMCQ, that were used by Karalunas et al. to identify the profiles, in 9-year-old children and found only little support for the initial theory-driven factorial model [33]. As Karalunas et al. [12, 25] conducted their analyses based on these subscale scores, this raises the question of whether different profiles might have been identified based on the individual TMCQ items.

Profiles identified based on community detection analysis also differed from the three main profiles that were previously identified by Martel et al. using latent profile analysis on a personality questionnaire in a large sample of children and adolescents with and without ADHD: “poor control”, “extraverted”, “introverted” [23]. While again the profiles could be mapped in some way (e.g., our first profile resembled the “poor control” group in their high levels of neuroticism and lower levels of openness and conscientiousness) there were also remarkable differences (e.g., whereas our first profile displayed intermediate levels of extraversion and agreeableness, the “poor control” group displayed the lowest levels on these factors).

To improve comparability with this previous study, we conducted a complementary analysis consisting of a latent profile analysis based on the BFQ-C items (see Supplement), and an optimal three-profile solution was identified. However, contrary to Karalunas et al. [25] who replicated their initial solution using both estimation techniques, here the profiles identified using latent profile analysis differed from the ones found using community detection. Based on latent profile analysis, the first profile was characterised by increased levels of ADHD severity and increased scores on all Big Five factors, and the second profile had decreased levels of neuroticism compared to the first. The third profile was the most stable between estimation approaches, and was characterized here by more normative values on all Big Five factor scores compared to the other two profiles. Hence, higher levels of neuroticism were this time identified in a single profile instead of two. In the present study, only the profiles identified with community detection were found to be clinically predictive. It should be noted that the estimation techniques are based on different inputs, as community detection uses the child-by-child correlations between scoring patterns, whereas the latent profile analysis uses the raw scores for each child. Potentially, this could make the community detection analysis less sensitive to the severity levels than latent profile analysis, and, therefore, both techniques could capture different information. Methodological studies are needed to disentangle the contributions of the two statistical clustering approaches.

Notably, the profiles identified based on latent profile analysis also differed from the “poor control”, “extraverted”, and “introverted” group identified in Martel et al. Lower levels of conscientiousness were found in two of our profiles, and higher levels of neuroticism in one profile instead of two [23]. Multiple methodological differences between this previous study and ours may explain these discrepant findings: (1) in Martel et al., groups were identified based on a sample including both healthy controls and children with ADHD; (2) ADHD cases included mixed ADHD presentations, whereas we only included combined-type cases; (3) our sample was characterised by a restricted age range (6–11) as opposed to the larger age range (6–18) in [23]; (4) recruitment strategies differed (community sample vs. clinically based). The possible effect of these factors on the identified communities should be examined in future studies.

When examining the predictive value of BFQ-C based profiles, we found that the identified personality profiles were a significant predictor of response to medication. This is a particularly relevant finding since, to date, predictors of treatment effect in ADHD have been elusive. More specifically, controlling for initial ADHD symptom severity, we found that children belonging to the third personality profile did not benefit from the use of medication treatment when compared to the other two groups. Children belonging to the third profile displayed more normative levels of neuroticism, agreeableness, and conscientiousness compared with the children in profile 1 and 2. Importantly, this finding did not appear to be confounded by group differences in initial ADHD severity, suggesting that personality profiles are unique predictors of medication effect. As profile 1 and 2 were both characterised by increased neuroticism when compared with profile 3, our finding is in line with previous research, which identified the irritability dimension to be of clinical predictive value [12, 25]. Personality measures are easily administered in the clinical setting, and our finding indicates that personality might be relevant to predict treatment response. Researchers trying to predict treatment responders vs. non-responders in ADHD based on machine learning could, therefore, potentially benefit from including a personality questionnaire among their measures [54]. Whether personality measures will also predict different responses to different types of treatments could further be investigated. Personality measures could ultimately become one of the tools used to provide personalized medicine to children with ADHD.

The profiles themselves were shown to be highly stable over time, whereas the profile membership of the children was much more variable. Notably, this result was similar to that reported by Karalunas et al. [25] where the profiles themselves were reproduced over three annual measurements but the membership stability varied between 36 and 66%.

Although at first, the moderate profile-membership stabilities might cast some doubt on the utility of these profiles for clinical practice, the high stabilities of the profiles themselves are clinically promising. Specifically, even though children might not be assigned to the same group over time, the finding that the profiles is stable over time suggests that there are some stable constellations of personality profiles among children with ADHD. This in itself can have clinical utility, as certain constellations of personality profiles might be predictive of clinical outcomes. It is then this ‘state’ that is predictive of clinical outcome rather than the stability of the state. This idea is supported by Karalunas et al. [25] in which they found that belonging to the irritable profile at any point rather than belonging to the irritable profile at all points was predictive for clinical outcomes. The stability of the profiles is especially noteworthy as the children grew older and some started the use of medication. While this might have affected the profile membership of the children, the profiles themselves were stable regardless of these changes. This again suggests that certain constellations of personality profiles are robust across time.

One potential limitation of our study as well as the previous ones (Karalunas et al. 12, 25) is the use of a single parent informant for most cases (91.6%), who could either be the father or the mother of the child. High mother–father agreement for higher order child personality traits has been reported in previous research, with Pearson correlations indexing mother–father agreement ranging from 0.54 for agreeableness and neuroticism to 0.77 for conscientiousness [55]. Nevertheless, this agreement was not complete, and presence of informant discrepancies might create confusion in research and clinical settings when utilizing ratings to predict later behaviour or to guide assessment and treatment. Presence of informant discrepancies could reflect in some cases underlying conflict in the family system, or the fact that mothers and fathers differentially monitor and evoke some relevant attributes of their child [55]. In separated couples, relevance of each parent’s evaluation could also depend on the time they spend with the child based on the custody schedule. In community detection analyses, informant discrepancies could result in different subgroup classifications for borderline individual cases, depending on whether father or mother reports are used. Future studies should, therefore, collect both father and mother ratings, to compute classification agreement rates, or at least collect data for all cases from the same informant. Another limitation is that there was no control sample, so that we could not compare our identified communities in the ADHD sample to those in a control sample. Yet, this did not affect our main objective to compare ADHD profiles identified using personality measures to those that were identified using temperament measures.

## Conclusions

In this study, we examined whether an irritable ADHD profile could be identified in a new sample based on personality dimensions. The identified profiles did only partially replicate the temperament-based profiles previously reported, as higher levels of neuroticism were found in two of thee detected profiles. Nonetheless, similarly to previous research, differentiating profiles based on current levels of emotional instability was associated with unique predictive value, as belonging to one of the two groups with high levels on this dispositional trait predicted a better response to medication treatment. Personality questionnaires might ultimately serve to provide more personalized medicine to children with ADHD. Other replication studies using both temperament and personality questionnaires will be needed to clarify whether these divergent findings are due to differences in sample characteristics vs. measurement instruments. Finally, although we did not replicate the ADHD profiles across measures of dispositional traits, it might turn out that both the profiles identified using temperament as well as the profiles identified using personality measures capture unique clinical predictive value.

## Electronic supplementary material

Below is the link to the electronic supplementary material.

Supplementary file1 (DOCX 37715 kb)

## Footnotes

1 We repeated the community detection analysis based on the item scores of the BFQ-C at T0 with single informant data instead of double informant data for the 15 participants who had both mother and father data available. Community outputs and individual classifications remained unchanged.

## References

[1] Benjamins JS, Migliorati F, Dekker K, Wassing R, Moens S, Blanken TF (2017). Insomnia heterogeneity: characteristics to consider for data-driven multivariate subtyping. Sleep Med Rev.

[2] Lombardo MV, Lai M-C, Baron-Cohen S (2019). Big data approaches to decomposing heterogeneity across the autism spectrum. Mol Psychiatry.

[3] Luo Y, Weibman D, Halperin JM, Li X (2019). A review of heterogeneity in attention deficit/hyperactivity disorder (ADHD). Front Hum Neurosci.

[4] Masi A, DeMayo MM, Glozier N, Guastella AJ (2017). An overview of autism spectrum disorder, heterogeneity and treatment options. Neurosci Bull.

[5] Milaneschi Y, Lamers F, Peyrot WJ, Abdellaoui A, Willemsen G, Hottenga J-J (2016). Polygenic dissection of major depression clinical heterogeneity. Mol Psychiatry.

[6] Rink L, Pagel T, Franklin J, Baethge C (2016). Characteristics and heterogeneity of schizoaffective disorder compared with unipolar depression and schizophrenia—a systematic literature review and meta-analysis. J Affect Disord.

[7] Reale L, Bartoli B, Cartabia M, Zanetti M, Costantino MA, Canevini MP (2017). Comorbidity prevalence and treatment outcome in children and adolescents with ADHD. Eur Child Adolesc Psychiatry.

[8] Arnold LE, Hodgkins P, Kahle J, Madhoo M, Kewley G (2015). Long-term outcomes of ADHD: academic achievement and performance. J Atten Disord.

[9] Fleck K, Jacob C, Philipsen A, Matthies S, Graf E, Hennighausen K (2015). Child impact on family functioning: a multivariate analysis in multiplex families with children and mothers both affected by attention-deficit/hyperactivity disorder (ADHD). Atten Defic Hyperact Disord.

[10] Schachar R (2014). Genetics of attention deficit hyperactivity disorder (ADHD): recent updates and future prospects. Curr Dev Disord Rep.

[11] Albajara Sáenz A, Villemonteix T, Massat I (2018) Structural and functional neuroimaging in attention-deficit/hyperactivity disorder. Dev Med Child Neurol. 10.1111/dmcn.14050 30276811

[12] Karalunas SL, Fair D, Musser ED, Aykes K, Iyer SP, Nigg JT (2014). Subtyping attention-deficit/hyperactivity disorder using temperament dimensions: toward biologically based nosologic criteria. JAMA Psychiatry.

[13] Fair DA, Bathula D, Nikolas MA, Nigg JT (2012). Distinct neuropsychological subgroups in typically developing youth inform heterogeneity in children with ADHD. PNAS.

[14] Sjowall D, Roth L, Lindqvist S, Thorell LB (2013). Multiple deficits in ADHD: executive dysfunction, delay aversion, reaction time variability, and emotional deficits. J Child Psychol Psychiatry.

[15] Karalunas SL, Gustafsson HC, Dieckmann NF, Tipsord J, Mitchell SH, Nigg JT (2017). Heterogeneity in development of aspects of working memory predicts longitudinal attention deficit hyperactivity disorder symptom change. J Abnorm Psychol.

[16] American Psychiatric Association (2013) Diagnostic and statistical manual of mental disorders 5. Washingtown D.C.

[17] Nigg JT (2006). Temperament and developmental psychopathology. J Child Psychol Psychiatry.

[18] Tackett JL, Kushner SC, Herzhoff K, Smack AJ, Reardon KW (2014). Viewing relational aggression through multiple lenses: temperament, personality, and personality pathology. Dev Psychopathol.

[19] Gomez R, Corr PJ (2014). ADHD and personality: a meta-analytic review. Clin Psychol Rev.

[20] Martel MM, Nigg JT, von Eye A (2009). How do trait dimensions map onto ADHD symptom domains?. J Abnorm Child Psychol.

[21] Nigg JT, John OP, Blaskey LG, Huang-Pollock CL, Willcutt EG, Hinshaw SP (2002). Big five dimensions and ADHD symptoms: links between personality traits and clinical symptoms. J Pers Soc Psychol.

[22] Nigg JT, Goldsmith HH, Sachek J (2004). Temperament and attention deficit hyperactivity disorder: the development of a multiple pathway model. J Clin Child Adolesc Psychol.

[23] Martel MM, Goth-Owens T, Martinez-Torteya C, Nigg JT (2010). A person-centered personality approach to heterogeneity in attention-deficit/hyperactivity disorder (ADHD). J Abnorm Psychol.

[24] Rubinov M, Sporns O (2011). Weight-conserving characterization of complex functional brain networks. Neuroimage.

[25] Karalunas SL, Gustafsson HC, Fair D, Musser ED, Nigg JT (2018). Do we need an irritable subtype of ADHD? Replication and extension of a promising temperament profile approach to ADHD subtyping. Psychol Assess.

[26] Brotman MA, Kircanski K, Stringaris A, Pine DS, Leibenluft E (2017). Irritability in youths: a translational model. Am J Psychiatry.

[27] Copeland WE, Brotman MA, Costello EJ (2015). Normative irritability in youth: developmental findings from the great smoky mountain study. J Am Acad Child Adolesc Psychiatry.

[28] Eyre O, Langley K, Stringaris A, Leibenluft E, Collishaw S, Thapar A (2017). Irritability in ADHD: associations with depression liability. J Affect Disord.

[29] Geller B, Zimerman B, Williams M, Delbello MP, Bolhofner K, Craney JL (2002). DSM-IV mania symptoms in a prepubertal and early adolescent bipolar disorder phenotype compared to attention-deficit hyperactive and normal controls. J Child Adolesc Psychopharmacol.

[30] Mick E, Spencer T, Wozniak J, Biederman J (2005). Heterogeneity of irritability in attention-deficit/hyperactivity disorder subjects with and without mood disorders. Biol Psychiatry.

[31] Waldman ID, Rowe R, Boylan K, Burke JD (2018) External validation of a bifactor model of oppositional defiant disorder. Mol Psychiatry. https://www.nature.com/articles/s41380-018-0294-z 10.1038/s41380-018-0294-z PMC6814504 30538308

[32] Whelan YM, Stringaris A, Maughan B, Barker ED (2013). Developmental continuity of oppositional defiant disorder subdimensions at ages 8, 10, and 13 years and their distinct psychiatric outcomes at age 16 years. J Am Acad Child Adolesc Psychiatry.

[33] Kotelnikova Y, Olino TM, Klein DN, Mackrell SVM, Hayden EP (2017). Higher- and lower-order factor analyses of the temperament in middle childhood questionnaire. Assessment.

[34] Rothbart MK (2011) Becoming who we are: temperament and personality in development. Guilford Press, pp 337

[35] Denissen JJA, van Aken MAG, Penke L, Wood D (2013). Self-Regulation underlies temperament and personality: an integrative developmental framework. Child Dev Perspect.

[36] De Pauw SSW (2017) Childhood personality and temperament. The Oxford Handbook of the Five Factor Model [Internet]. 2017 May 27 [cited 2019 Jan 24]; Available from: https://www.oxfordhandbooks.com/view/10.1093/oxfordhb/9780199352487.001.0001/oxfordhb-9780199352487-e-21

[37] Rothbart MK, Posner MI (2005). Genes and experience in the development of executive attention and effortful control. New Dir Child Adolesc Dev.

[38] Shiner RL, DeYoung CG (2013) The structure of temperament and personality traits: a developmental perspective. In: The Oxford handbook of developmental psychology, Vol 2: Self and other. Oxford University Press; New York, pp 113–41 (Oxford library of psychology).

[39] Zohar AH, Zwir I, Wang J, Cloninger CR, Anokhin AP (2019). The development of temperament and character during adolescence: the processes and phases of change. Dev Psychopathol.

[40] Barbaranelli C, Caprara GV, Rabasca A, Pastorelli C (2003). A questionnaire for measuring the big five in late childhood. Personal Individ Differ.

[41] del Barrio V, Carrasco MÁ, Holgado FP (2006). Factor structure invariance in the children’s big five questionnaire. Eur J Psychol Assess.

[42] Holgado-Tello FP, Carrasco-Ortiz MÁ, del Barrio-Gándara MV, Chacón-Moscoso S (2009). Factor analysis of the big five questionnaire using polychoric correlations in children. Qual Quant.

[43] Olivier M, Herve M (2015). The big five questionnaire for children (BFQ-C): a french validation on 8- to 14-year-old children. Personal Individ Differ.

[44] Kaufman J, Birmaher B, Brent D, Rao U, Flynn C, Moreci P (1997). Schedule for affective disorders and schizophrenia for school-age children-present and lifetime version (K-SADS-PL): initial reliability and validity data. J Am Acad Child Adolesc Psychiatry.

[45] DuPaul GJ, Power TJ, Anastopoulos AD, Reid R (1998) ADHD rating scale—IV: checklists, norms, and clinical interpretation. Guilford Press; New York, pp 79 (ADHD Rating Scale—IV: Checklists, norms, and clinical interpretation).

[46] Goodman R (1999). The extended version of the strengths and difficulties questionnaire as a guide to child psychiatric caseness and consequent burden. J Child Psychol Psychiatry.

[47] Goodman R (2001). Psychometric properties of the strengths and difficulties questionnaire. J Am Acad Child Adolesc Psychiatry.

[48] Newman MEJ (2006). Modularity and community structure in networks. Proc Natl Acad Sci USA.

[49] Newman MEJ, Girvan M (2004). Finding and evaluating community structure in networks. Phys Rev E.

[50] Capdevila-Brophy C, Artigas-Pallarés J, Navarro-Pastor JB, García-Nonell K, Rigau-Ratera E, Obiols JE (2014). ADHD predominantly inattentive subtype with high sluggish cognitive tempo: a new clinical entity?. J Atten Disord.

[51] Sussman TJ, Posner J (2019). Editorial: neural correlates of sluggish cognitive tempo: biological evidence of a distinct clinical entity?. J Am Acad Child Adolesc Psychiatry.

[52] Gaias LM, Gartstein MA, Fisher PA, Putnam SP, Räikkönen K, Komsi N (2012). Cross-cultural temperamental differences in infants, children, and adults in the United States of America and Finland. Scand J Psychol.

[53] Sung J, Beijers R, Gartstein MA, de Weerth C, Putnam SP (2015). Exploring temperamental differences in infants from the United States of America (US) and the Netherlands. Eur J Dev Psychol.

[54] Wong HK, Tiffin PA, Chappell MJ, Nichols TE, Welsh PR, Doyle OM, et al. (2017) Personalized medication response prediction for attention-deficit hyperactivity disorder: learning in the model space vs. learning in the data space. Front Physiol 8:199. 10.3389/fphys.2017.00199 PMC5387107 28443027

[55] Tackett JL (2011). Parent informants for child personality: agreement, discrepancies, and clinical utility. J Pers Assess.

