# Supplementary material for: Is an irritable ADHD profile traceable using personality dimensions? Replicability, stability, and predictive value over time of data-driven profiles
Source: Eur Child Adolesc Psychiatry. 2020 May 12;30(4):633–45. doi: 10.1007/s00787-020-01546-z (PMC8041702; doi:10.1007/s00787-020-01546-z)
Supplement: Supplementary file 1 — Supplementary file1 (DOCX 37715 kb) [file 787_2020_1546_MOESM1_ESM.docx]

**Supplementary Material**

*Is an irritable ADHD profile traceable using personality dimensions? Replicability, stability, and predictive value over time of data-driven profiles.*

Tessa F Blanken, Ophélie Courbet, Nathalie Franc, Ariadna Albajara Sáenz, Eus Van Someren, Philippe Peigneux, Thomas Villemonteix

**Supplementary Methods and Results**

**1. Exploratory factor analysis**

To interpret the communities at a more detailed level, we conducted an exploratory factor analysis on the BFQ-C items. First we conducted a principal component analysis on all 65 items simultaneously. The five-factor factorial solution explained 41.1% of the total variance, similar to the original validation of the BFQ-C in which the five-factor solution explained 38.3% of the variance (Olivier & Hervé, 2015). To investigate the personality factors at a more detailed level we subsequently performed a principal component analysis on the items of each personality factor separately. We inspected the screen plot and the number of components with variance larger than 1: 3 for Neuroticism, 4 for Conscientiousness, 5 for Agreeableness, and 6 for Extraversion and Openness. To be consistent across the personality factors, we choose to inspect the three-factor factorial solution for all personality factors. For all five personality factors, the three-factor solution explained more than 50% of the variance, ranging from 53.1% for Extraversion to 60.7% for Openness. Therefore we choose the three-factorial solution to interpret the identified communities at a more detailed level. Supplementary Table S1.

**References**

Olivier, M., & Herve, M. (2015). The Big Five Questionnaire for Children (BFQ-C): A French validation on 8- to 14-year-old children. *Personality and Individual Differences*, *87*, 55–58. https://doi.org/10.1016/j.paid.2015.07.030

**Supplementary Figure S1**

Profiles identified based on the scores of the five personality factors: Extraversion, Agreeableness, Conscientiousness, Neuroticism, and Openness.

**Supplementary Table S1: Exploratory Factor Analysis loadings**

| Items | Facets | | |
| --- | --- | --- | --- |
| *Extraversion* | gregariousness | dominance | open to others |
| 1. I like to spend time with other people | **0.85** | -0.04 | -0.07 |
| 9. I like to compete | 0.13 | **0.60** | 0.00 |
| 14. I like to be active | 0.19 | **0.54** | 0.13 |
| 19. I like to be around others | **0.84** | 0.04 | -0.05 |
| 23. I can tell others what I think | 0.05 | -0.03 | **0.82** |
| 26. I say what I think | -0.06 | 0.02 | **0.86** |
| 35. I find things to do so I am not bored | 0.13 | -0.64 | 0.26 |
| 40. I like to talk with others | **0.64** | 0.06 | 0.26 |
| 42. I am able to get people to agree with me | -0.09 | **0.52** | 0.37 |
| 50. Others listen and do what I say | 0.27 | **0.37** | 0.07 |
| 55. I like to joke around | 0.21 | -0.04 | **0.40** |
| 57. I make friends easily | **0.74** | 0.00 | -0.02 |
| 63. I am happy and active | **0.56** | -0.02 | 0.10 |
|  |  |  |  |
| *Agreeableness* | kindness | trust | sharing |
| 2. I share my things | 0.03 | 0.09 | **0.85** |
| 11. I am honest and kind | **0.83** | 0.05 | -0.13 |
| 13. I know when others need my help | **0.64** | -0.10 | 0.22 |
| 16. I like to give gifts | **0.52** | -0.16 | 0.25 |
| 21. I forgive | -0.13 | **0.70** | 0.19 |
| 27. I am nice to all my classmates | **0.60** | 0.03 | 0.27 |
| 32. I treat others with kindness | **0.54** | 0.16 | 0.34 |
| 38. I am polite when I talk to others | **0.67** | 0.22 | -0.23 |
| 45. I help classmates when they have trouble | **0.42** | -0.04 | 0.31 |
| 47. I trust others | 0.04 | **0.47** | 0.38 |
| 51. I treat even people I dislike with kindness | 0.09 | **0.79** | -0.11 |
| 60. I think people are good and honest | 0.09 | **0.69** | 0.06 |
| 64. I let other people use my things | 0.03 | 0.02 | **0.87** |
|  |  |  |  |
| *Conscientiousness* | self discipline | orderliness | persever-ance |
| 3. I do my work carefully | 0.22 | **0.45** | 0.39 |
| 7. I enjoy working hard | **0.78** | -0.03 | 0.01 |
| 20. I get involved and do my best | 0.41 | -0.06 | **0.49** |
| 22. I concentrate in class | **0.65** | -0.13 | 0.12 |
| 25. I check my homework many times | **0.53** | 0.21 | -0.06 |
| 28. I respect and follow rules | **0.68** | 0.01 | -0.16 |
| 34. I keep my appointments | 0.27 | 0.29 | **0.40** |
| 37. My room is neat and organized | 0.06 | **0.84** | -0.16 |
| 44. I have to finish what I start | -0.11 | 0.19 | **0.82** |
| 48. I keep my school things neat and organized | 0.00 | **0.73** | 0.27 |
| 53. I finish homework before I play | **0.47** | 0.14 | -0.01 |
| 56. I pay attention to what I am doing | 0.11 | -0.28 | **0.72** |
| 65. I take care of my responsibilities | **0.69** | 0.12 | -0.02 |
|  |  |  |  |
| *Neuroticism* | irritability | impatience | sad anxious |
| 4. I get nervous | **0.77** | 0.01 | 0.07 |
| 6. I am in a bad mood | **0.62** | -0.21 | 0.26 |
| 8. I get into heated arguments | **0.69** | 0.27 | -0.09 |
| 15. I get angry easily | **0.81** | -0.02 | 0.12 |
| 17. I argue with others | **0.72** | -0.11 | -0.03 |
| 29. My feelings get hurt easily | 0.35 | 0.09 | **0.37** |
| 31. I am sad | 0.11 | -0.11 | **0.75** |
| 39. I have to do things immediately | -0.11 | **0.76** | 0.13 |
| 41. I am not patient | 0.19 | **0.77** | -0.02 |
| 49. I lose my calm easily | **0.82** | 0.10 | -0.08 |
| 54. I get irritated with difficult things | 0.45 | **0.48** | -0.02 |
| 58. I cry | 0.25 | -0.13 | **0.58** |
| 61. I worry about silly things | -0.12 | 0.32 | **0.74** |
|  |  |  |  |
| *Openness* | intellectual capacity | creativity | curiosity |
| 5. I know a lot of things | 0.43 | -0.07 | **0.49** |
| 10. I daydream a lot | 0.10 | **0.70** | 0.12 |
| 12. It is easy for me to learn | **0.87** | -0.03 | -0.08 |
| 18. I am able to give correct answers | **0.79** | 0.02 | 0.03 |
| 24. I like to read books | **0.54** | 0.18 | 0.13 |
| 30. I understand directions immediately | **0.88** | 0.05 | -0.07 |
| 33. I like scientific TV shows | 0.14 | 0.12 | **0.67** |
| 36. I like to watch the news and know what is going on | -0.14 | -0.02 | **0.82** |
| 43. I make up new games and things to do | -0.06 | **0.89** | -0.05 |
| 46. I am able to solve math problems | **0.67** | -0.09 | -0.01 |
| 52. I like to learn new things | 0.26 | 0.07 | **0.57** |
| 59. would like to travel, learn of other countries | -0.06 | 0.00 | **0.74** |
| 62. I understand things immediately | **0.72** | -0.05 | 0.18 |

Exploratory factor loadings. All loadings ≥ 0.40 are shown in bold font.

**Supplementary Table S2: Mean (±SD) on the Big Five personality factor scores for the three ADHD profiles identified using community detection compared with a previous normative sample.**

| **Characteristics** | **Normative sample** | **Type 1** | **Type 2** | **Type 3** | **F(3,349)** | **Post-hoc** |
| --- | --- | --- | --- | --- | --- | --- |
| Big Five Personality Scores | | | | | | |
| Extraversion | 2.81 (0.53) | 2.55 (0.45) | 2.39 (0.52) | 2.68 (0.56) | 11.69 *** | Norm>1,2 |
|  |  |  |  |  |  |  |
| Agreeableness | 2.74 (0.53) | 2.50 (0.50) | 1.82 (0.50) | 2.81 (0.49) | 62.97 *** | Norm>1,2 |
|  |  |  |  |  |  |  |
| Neuroticism | 1.63 (0.80) | 3.07 (0.38) | 3.18 (0.40) | 2.44 (0.56) | 123.69 *** | Norm>1,2,3 |
|  |  |  |  |  |  |  |
| Consciousness | 2.41 (0.66) | 1.11 (0.56) | 1.15 (0.52) | 1.42 (0.61) | 109. 37 *** | Norm>1,2,3 |
|  |  |  |  |  |  |  |
| Openness to Experience | 2.77 (0.57) | 1.82 (0.60) | 2.73 (0.62) | 2.52 (0.72) | 26.31 *** |  |
|  |  |  |  |  |  |  |

**Supplementary Table S3: Mean (±SD) on the Big Five personality factors and EFA subscales for the three profiles identified using community detection.**

| **Characteristics** | **Time** | **Type 1** | **Type 2** | **Type 3** | **F(2,175)** | **Post-hoc** |
| --- | --- | --- | --- | --- | --- | --- |
| Big Five Personality Scores | | | | | | |
| Extraversion | T0 | 2.55 (0.45) | 2.39 (0.52) | 2.68 (0.56) | 5.65* | 3>2 |
|  | T1 | 2.28 (0.52) | 2.40 (0.54) | 2.74 (0.45) | 12.45* | 3>1,2 |
| Agreeableness | T0 | 2.50 (0.50) | 1.82 (0.50) | 2.81 (0.49) | 71.39* | 3>1>2 |
|  | T1 | 2.65 (0.37) | 2.05 (0.45) | 2.90 (0.48) | 66.62* | 3>1>2 |
| Neuroticism | T0 | 3.07 (0.38) | 3.18 (0.40) | 2.44 (0.56) | 48.60* | 1,2>3 |
|  | T1 | 3.10 (0.43) | 3.07 (0.43) | 2.12 (0.57) | 76.72* | 1,2>3 |
| Consciousness | T0 | 1.11 (0.56) | 1.15 (0.52) | 1.42 (0.61) | 5.49* | 3>1,2 |
|  | T1 | 1.31 (0.64) | 1.24 (0.54) | 1.64 (0.63) | 8.10* | 3>1,2 |
| Openness to Experience | T0 | 1.82 (0.60) | 2.73 (0.62) | 2.52 (0.72) | 30.23* | 2,3>1 |
|  | T1 | 2.11 (0.60) | 2.88 (0.52) | 2.81 (0.66) | 23.30* | 2,3>1 |
| Big Five Personality Subscales | | | | | | |
| Gregariousness | T0 | 2.85 (0.87) | 2.41 (0.84) | 3.15 (0.72) | 15.02* | 1,3>2 |
|  | T1 | 2.60 (0.64) | 2.39 (0.63) | 3.22 (0.55) | 32.29* | 3>1,2 |
| Open to others | T0 | 3.02 (0.81) | 2.91 (0.86) | 3.09 (0.77) | 0.82 |  |
|  | T1 | 2.48 (0.94) | 2.72 (0.87) | 2.75 (0.82) | 1.27 |  |
| Dominance | T0 | 1.82 (0.69) | 2.19 (0.77) | 1.86 (0.84) | 4.33* | 2>1,3 |
|  | T1 | 1.85 (0.64) | 2.41 (0.75) | 2.29 (0.70) | 7.66* | 2,3>1 |
| Kindness | T0 | 2.52 (0.59) | 1.99 (0.65) | 2.91 (0.61) | 39.25* | 3>1>2 |
|  | T1 | 2.76 (0.54) | 2.17 (0.53) | 3.04 (0.56) | 42.01* | 3>1>2 |
| Trust | T0 | 2.38 (0.74) | 1.56 (0.59) | 2.57 (0.69) | 44.54* | 1,3>2 |
|  | T1 | 2.48 (0.61) | 1.80 (0.78) | 2.63 (0.67) | 24.39* | 1,3>2 |
| Sharing | T0 | 2.68 (0.87) | 1.72 (0.92) | 2.90 (0.82) | 34.74* | 1,3>2 |
|  | T1 | 2.64 (0.84) | 1.91 (0.72) | 2.95 (0.88) | 27.08* | 1,3>2 |
| Self-discipline | T0 | 0.90 (0.62) | 0.88 (0.54) | 1.20 (0.62) | 5.97* | 3>1,2 |
|  | T1 | 1.19 (0.58) | 1.01 (0.54) | 1.54 (0.69) | 12.24* | 3>1,2 |
| Perseverance | T0 | 1.43 (0.94) | 1.61 (0.94) | 1.64 (0.93) | 0.62 |  |
|  | T1 | 1.47 (1.09) | 1.75 (0.84) | 1.84 (0.94) | 1.90 |  |
| Orderliness | T0 | 0.82 (0.76) | 0.88 (0.79) | 1.28 (0.91) | 5.48* | 3>1,2 |
|  | T1 | 1.15 (0.91) | 0.81 (0.87) | 1.30 (0.79) | 5.66* | 3>2 |
| Irritability | T0 | 3.09 (0.42) | 3.29 (0.44) | 2.17 (0.72) | 75.93* | 1,2>3 |
|  | T1 | 3.05 (0.55) | 3.10 (0.53) | 1.85 (0.78) | 72.96* | 1,2>3 |
| Sad/anxious | T0 | 2.58 (0.68) | 2.71 (0.68) | 2.18 (0.81) | 9.55* | 1,2>3 |
|  | T1 | 2.73 (0.75) | 2.44 (0.75) | 1.73 (0.79) | 24.52* | 1,2>3 |
| Impatience | T0 | 3.71 (0.43) | 3.57 (0.53) | 3.31 (0.64) | 7.14* | 1,2>3 |
|  | T1 | 3.36 (0.50) | 3.41 (0.47) | 2.55 (0.64) | 46.40* | 1,2>3 |
| Intellectual capacity | T0 | 1.51 (0.74) | 2.69 (0.80) | 2.53 (0.84) | 28.87* | 2,3>1 |
|  | T1 | 1.87 (0.68) | 2.85 (0.67) | 2.68 (0.85) | 21.70* | 2,3>1 |
| Curiosity | T0 | 1.84 (0.80) | 2.71 (0.79) | 2.77 (0.78) | 19.48* | 2,3>1 |
|  | T1 | 2.18 (0.94) | 2.91 (0.68) | 2.90 (0.85) | 11.56* | 2,3>1 |
| Creativity | T0 | 2.72 (0.88) | 2.87 (0.88) | 2.93 (0.88) | 0.69 |  |
|  | T1 | 2.63 (1.01) | 2.88 (0.84) | 2.96 (0.90) | 1.64 |  |

**2. Ancillary analysis: Latent Profile Analysis**

In the current study we have used community detection to identify data-driven profiles of ADHD. Alternatively, latent profile analysis (LPA) could be used to identify data-driven classes. For personality in children and adolescents with ADHD, this has been done in a previous study that used the Big five as a dispositional framework (Martel et al. 2010). Furthermore, Karalunas et al. (2018) replicated their temperament-based 3-profile solution obtained through community detection analysis with LPA. Hence, we also report on the optimal solution for a latent profile analysis based on the 65-items of the BFQ-C.

*Methods.* To identify the optimal model, we followed the procedures by Martel et al. (2010) and estimated all one to seven class solutions, and determining the optimal model by minimizing the BIC. We performed the latent profile analysis on all 65 items using Latent GOLD 5.0 (Vermunt & Magidson, 2016).

*Personality profiles*. LPA optimally identified 3 classes, of 67 (38%), 65 (37%) and 46 (25%) children respectively, see Table S4. The identified profiles are visualized in the Supplementary Figure S2, and the descriptive information is given in the Supplementary Table S5. In terms of personality features, the first two profiles were both characterized by low levels of extraversion, agreeableness and conscientiousness, while differing on neuroticism (profile 1 > profile 2). In comparison, profile 3 displayed scores closer to the normative controls on all Big Five factors, and may therefore be considered as a “resilient group”.

Clinically, profile 1 children displayed higher global and inattentive scores at the ADHD-RS than the other two profiles. Profile 1 and 2 also had higher hyperactive/impulsive scores compared to profile 3.

Of note, children were differently distributed to the LPA-based profiles versus the community-based profiles. Specifically, LPA-based profile 1 (67 children) included a mixed sample of children from each profile identified with community detection analysis (profile 1: 24 children; profile 2: 36 children; profile 3: 7 children). The same was true for LPA-based profile 2 (65 children; profile 1: 11 children; profile 2: 30 children; profile 3: 24 children). Finally, profile 3 (46 children) was the most stable, including mostly children from our original Profile 3 (profile 1: 3 children; profile 2: 7 children; profile 3 : 36 children). Hence, it appears that the first two profiles identified using community detection analysis were reorganized through latent profile analysis, differing now on ADHD severity and neuroticism rather than agreeableness and openness.

Clinical Prediction. The latent class memberships did not predict ADHD severity at follow-up over and above baseline ADHD-RS total score, baseline SDQ Impact score, age, and sex. Specifically, the decrease in ADHD-RS total score for children in class 1 (-5.9±8.1 mean±SD), class 2 (-6.1±8.7 mean±SD, t = -0.94, p = 0.35) and class 3 (-2.8±7.9 mean±SD, t = 1.24, p = 0.22) did not differ.

Only medication use was predictive of ADHD-RS total score at follow-up (t = -1.80, p = 0.05). Start of medication use over one-year follow-up did not differ across classes (p=0.42), and we did not find an interaction between LPA membership and medication effect (t < 1.11, p > 0.26)

In conclusion, the LPA-based classes capture somewhat different profiles and seem less clinically informative.

**References**

Martel, M.M., Goth-Owens, T., Martinez-Torteya, C., Nigg, J.T. A Person-Centered Personality Approach to Heterogeneity in Attention-Deficit/Hyperactivity Disorder (ADHD). *J Abnorm Psychol*. 2010 Feb;119(1):186–96

Vermunt, J.K., & Magidson J. Technical guide for Latent GOLD 5.0: Basic, Advanced, and Syntax. *Belmont, MA: Statistical Innovations Inc* 2016.

**Supplementary Table S4: Latent profile analysis fit indices for 1 to 7 classes based on 65 items.**

|  | **Loglikelihood** | **AIC** | **BIC** | **Entropy** |
| --- | --- | --- | --- | --- |
| 1-profile | -17109.38 | 34478.77 | 34892.40 |  |
| 2-profile | -16395.54 | 33313.07 | 34143.52 | **5.59** |
| 3-profile | -15948.08 | 32680.17 | **33927.43** | 2.54 |
| 4-profile | -15680.03 | 32406.06 | 34070.14 | 1.50 |
| 5-profile | -15459.66 | 32227.31 | 34308.20 | 2.19 |
| 6-profile | -15263.72 | **32097.44** | 34595.14 | 2.42 |
| 7-profile | **-15211.98** | 32255.96 | 35170.47 | 1.26 |

**Supplementary Table S5: Demographics and mean (±SD) of the Big Five personality scores, comorbidities, and ADHD severity for the three profiles identified using latent profile analysis.**

| **Characteristics** | **Type 1** | **Type 2** | **Type 3** | **Test** | **Post-hoc (Tukey)** |
| --- | --- | --- | --- | --- | --- |
| Big Five Personality Scores |  |  |  |  |  |
| Extraversion | 2.48 (0.56) | 2.33 (0.40) | 2.89 (0.45) | *F*(2,105) = 19.42** | 3>1,2 |
| Agreeableness | 2.10 (0.66) | 2.10 (0.47) | 3.00 (0.45) | *F*(2,112) = 60.78** | 3>1,2 |
| Neuroticism | 3.21 (0.36) | 2.62 (0.57) | 2.75 (0.61) | *F*(2,97) = 29.78** | 1,3>2 |
| Consciousness | 1.03 (0.54) | 1.14 (0.42) | 1.71 (0.57) | *F*(2,104) = 22.55** | 3>1,2 |
| Openness to Experience | 2.37 (0.70) | 2.30 (0.68) | 3.05 (0.50) | *F*(2,115) = 28.35** | 3>1,2 |
| Basic demographics |  |  |  |  |  |
| N | 67 | 65 | 46 |  |  |
| (Boys: Girls) | (51: 16) | (49: 16) | (35: 11) | *χ*2(2) = 0.01 |  |
| Age mean (sd) years | 9.3 (1.2) | 8.1 (1.3) | 8.0 (1.6) | *F*(2,6) = 1.14 |  |
| Comorbidity N (%) |  |  |  |  |  |
| GAD | 14 (20.9) | 7 (10.8) | 9 (19.6) | *χ*2(2) = 2.74 |  |
| Specific phobia | 5 (7.5) | 2 (3.1) | 1 (2.2) | *χ*2(2) = 2.26 |  |
| SAD | 2 (3.0) | 1 (1.5) | 2 (4.3) | *χ*2(2) = 0.79 |  |
| Social Phobia | 1 (1.5) | 1 (1.5) | 0 (0.0) | *χ*2(2) = 0.71 |  |
| Enuresia | 2 (3.0) | 2 (3.1) | 1 (2.2) | *χ*2(2) = 0.09 |  |
| Encopresia | 0 (0.0) | 1 (1.5) | 1 (2.2) | *χ*2(2) = 1.32 |  |
| Tic Disorder | 3 (4.5) | 2 (3.1) | 4 (8.7) | *χ*2(2) = 1.85 |  |
| ODD | 6 (9.0) | 3 (4.6) | 1 (2.2) | χ2(2) = 2.56 |  |
| DMDD | 13 (19.4) | 8 (12.3) | 3 (6.5) | *χ*2(2) = 4.00 |  |
| ADHD and severity measures |  |  |  |  |  |
| ADHD-RS Total Score | 44.00 (6.27) | 39.55 (6.65) | 39.47 (6.33) | *F*(2,103) = 9.79** | 1>2,3 |
| ADHD-RS Inattentive Score | 22.16 (3.62) | 20.13 (3.88) | 19.13 (4.45) | *F*(2,103) = 8.83** | 1>2,3 |
| ADHD-RS Hyp/Imp Score | 21.88 (3.79) | 19.46 (4.47) | 20.39 (4.07) | *F*(2,103) = 5.55* | 1>2 |
| SDQ Impact Score | 6.65 (2.55) | 6.09 (2.57) | 4.91 (2.32) | *F*(2,107) = 6.87* | 1,2>3 |

*Note.* Hyp/Imp = Hyperactive/Impulsive; GAD = Generalised Anxiety Disorder; SAD = Separation Anxiety Disorder; ODD = Oppositional Defiant Disorder;

DMDD = Disruptive Mood Dysregulation Disorder; ADHD-RS = Attention-Deficit with Hyperactivity Rating Scale IV;

SDQ= Strengths & Difficulties Questionnaire.

* significant at *p* < .05

** significant at *p* <.001

**Supplementary Figure S2: Identified profiles and individual patterns using latent profile analysis.**


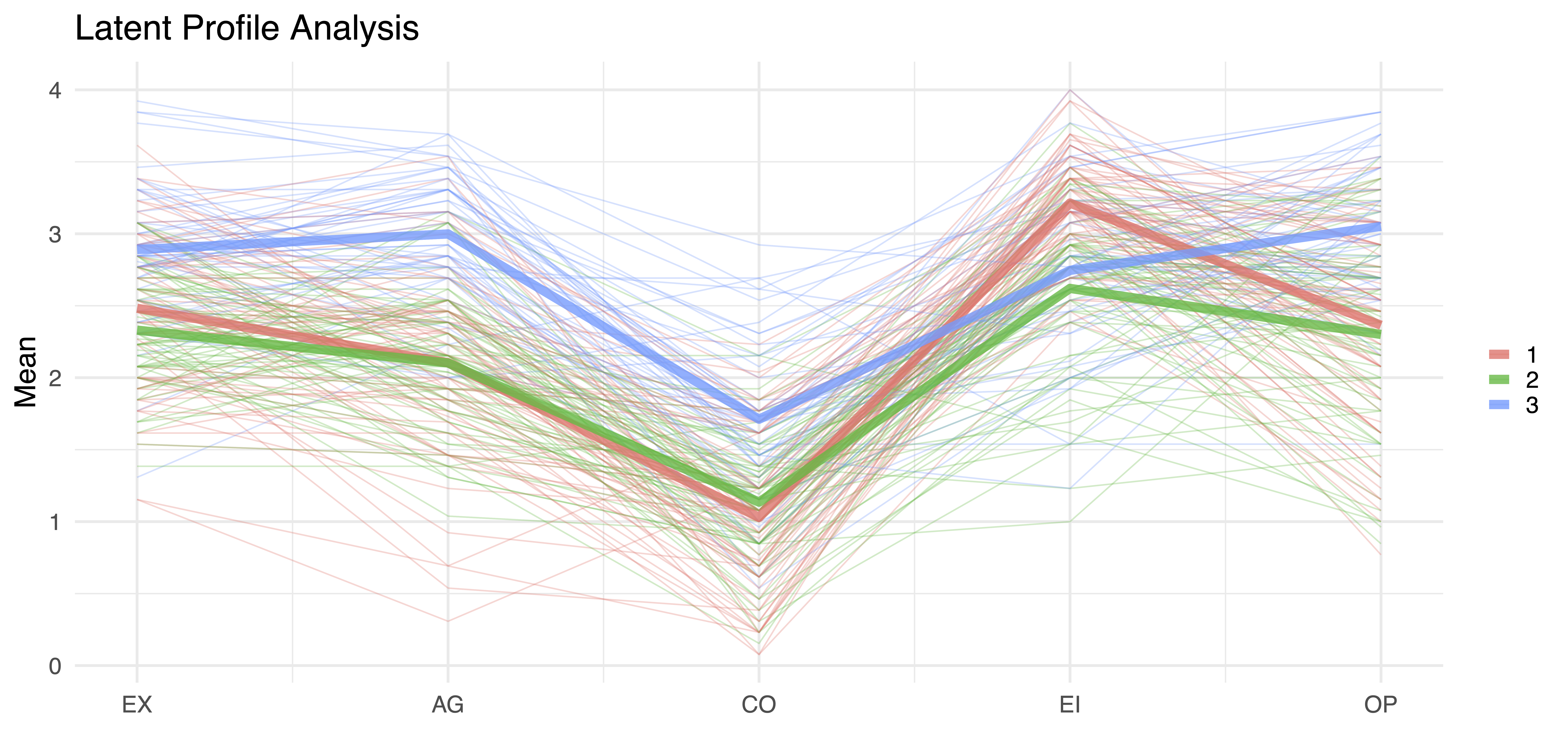


Note. Abbreviations: EX = Extraversion, AG = Agreeableness, CO = Conscientiousness, EI = Emotional Instability, OP = Openness.

**Supplementary Figure S3: Identified profiles and individual patterns using community detection.**


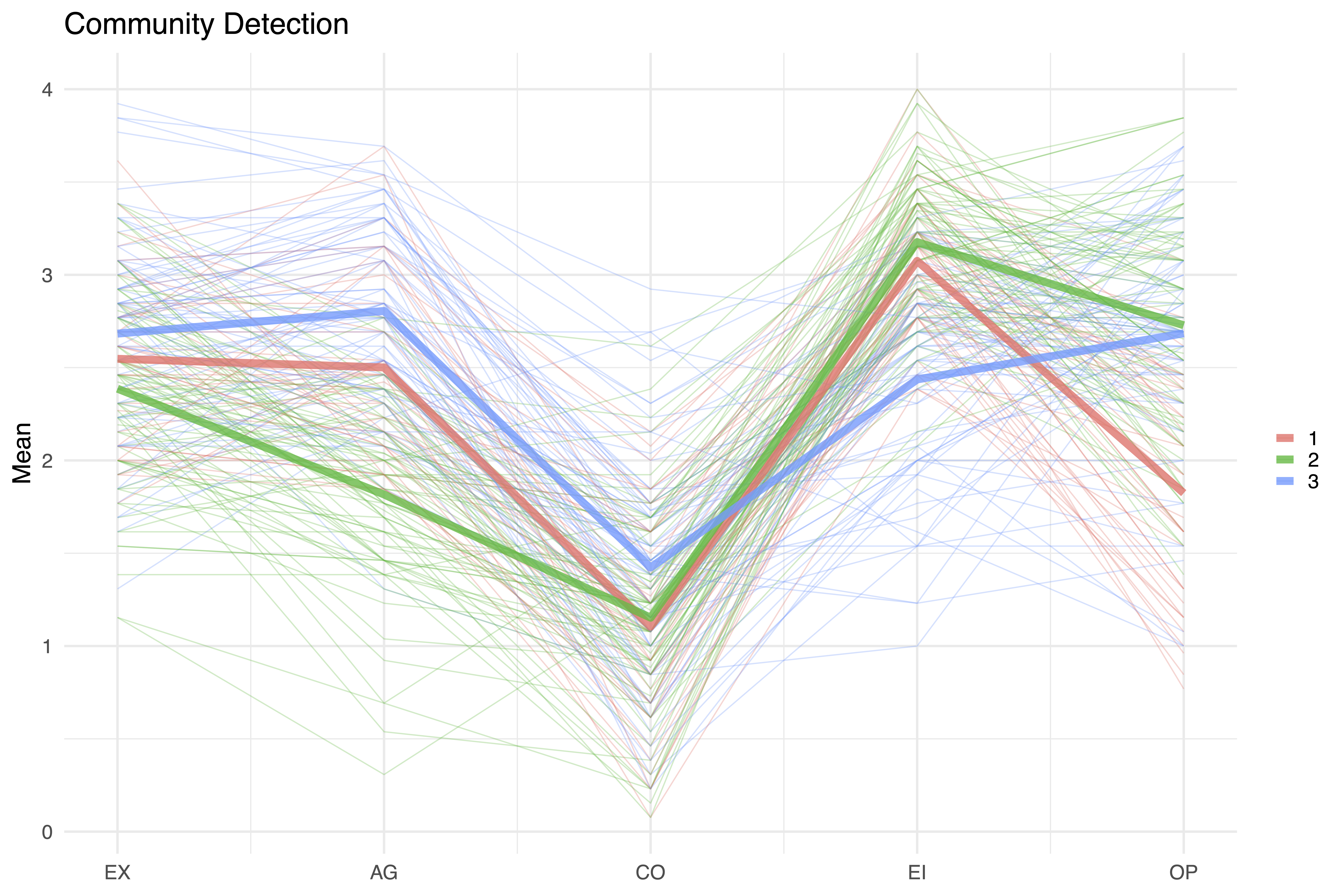


Note. Abbreviations: EX = Extraversion, AG = Agreeableness, CO = Conscientiousness, EI = Emotional Instability, OP = Openness.
